# Rapid SARS-CoV-2 Virus Enrichment and RNA Extraction for Efficient Diagnostic Screening of Pooled Nasopharyngeal or Saliva Samples for Dilutions Up to 1:100

**DOI:** 10.3390/diagnostics12061398

**Published:** 2022-06-06

**Authors:** Jamila S. Marshall, Rachelle Turiello, Larissa L. Cunha, Ella V. Frazier, Jeff Hickey, Jeff Chapman, Melinda D. Poulter, Heather L. Fehling, James P. Landers

**Affiliations:** 1 Department of Chemistry, University of Virginia, Charlottesville, VA 22904, USA; rat3a@virginia.edu (R.T.); llc9t@virginia.edu (L.L.C.); evf5mb@virginia.edu (E.V.F.); jpl5e@virginia.edu (J.P.L.); 2 MicroGEM International PLC, Charlottesville, VA 22903, USA; j.hickey@microgembio.com (J.H.); j.chapman@microgem.com (J.C.); 3 Department of Pathology, University of Virginia, Charlottesville, VA 22903, USA; mdp3s@virginia.edu; 4 Clinical Reference Laboratory, Lenexa, KS 66215, USA; heather.fehling@crlcorp.com; 5 Department of Mechanical and Aerospace Engineering, University of Virginia, Charlottesville, VA 22904, USA; 6 Department of Clinical Microbiology, University of Virginia, Charlottesville, VA 22903, USA

**Keywords:** SARS-CoV-2, COVID-19, sample pooling, Polymerase Chain Reaction (PCR), enzymatic extraction, nasopharyngeal (NP) swabs, saliva, nanoparticles

## Abstract

As COVID-19 transmission control measures are gradually being lifted, a sensitive and rapid diagnostic method for large-scale screening could prove essential for monitoring population infection rates. However, many rapid workflows for SARS-CoV-2 detection and diagnosis are not amenable to the analysis of large-volume samples. Previously, our group demonstrated a technique for SARS-CoV-2 nanoparticle-facilitated enrichment and enzymatic lysis from clinical samples in under 10 min. Here, this sample preparation strategy was applied to pooled samples originating from nasopharyngeal (NP) swabs eluted in viral transport medium (VTM) and saliva samples diluted up to 1:100. This preparation method was coupled with conventional RT-PCR on gold-standard instrumentation for proof-of-concept. Additionally, real-time PCR analysis was conducted using an in-house, ultra-rapid real-time microfluidic instrument paired with an experimentally optimized rapid protocol. Following pooling and extraction from clinical samples, average cycle threshold (C_T_) values from resultant eluates generally increased as the pooling dilution factor increased; further, results from a double-blind study demonstrated 100% concordance with clinical values. In addition, preliminary data obtained from amplification of eluates prepared by this technique and analyzed using our portable, ultra-rapid real-time microfluidic PCR amplification instrument showed progress toward a streamlined method for rapid SARS-CoV-2 analysis from pooled samples.

## 1. Introduction

The novel Betacoronavirus severe acute respiratory syndrome coronavirus 2 (SARS-CoV-2) was identified in Wuhan, China, in December 2019 and quickly spread throughout the world [1,2]. As of early January 2022, the coronavirus disease 2019 (COVID-19) pandemic has affected over 290 million people and caused more than 5.45 million deaths globally [3]. Several methods for sample collection and detection have emerged in recent months to address the increased demand for diagnostic laboratory testing and SARS-CoV-2 surveillance of asymptomatic persons, and to accommodate varying community needs [4,5]. In addition to simply diversifying testing methodologies available, it is important that all novel approaches for coronavirus clinical testing are amenable to simple implementation, are relatively inexpensive, and are not prohibitively time-consuming.

With regard to sample collection and cellular lysis, the focus of diagnostic testing for SARS-CoV-2 has been on symptomatic persons via testing of upper respiratory specimens collected by swabs and extracted by a solid-phase approach [4,5]. While nasopharyngeal (NP) specimens continue to be the COVID-19 laboratory diagnostic standard [6,7], saliva sampling has garnered significant attention as a low-cost, non-invasive alternative that affords comparable sensitivity for SARS-CoV-2 detection [8]. Furthermore, saliva specimens provide a more facile opportunity for pooled surveillance testing, which is a strategy proposed by several institutions for the monitoring of COVID-19 transmission [9,10]. Alternative viral lysis methods have also been demonstrated; notably, our group proposed a technique for SARS-CoV-2 nanoparticle-facilitated enrichment and enzymatic lysis from clinical samples that leverages existing PDQeX technology in under 10 min [11]. This method demonstrated comparable sensitivity to gold-standard methods for RNA isolation from clinical samples and provided positive diagnoses from NP specimens and saliva collections.

Beyond sample collection and following the lysis and isolation of viral RNA from swabs, reverse transcription-polymerase chain reaction (RT-PCR) is most commonly used for confirmatory detection and diagnosis of the virus. This approach is preferential over other molecular-based assays due to its capacity for sensitive and specific pathogen detection and potential for implementation with rapid diagnostic workflows [12].

Microfluidic alternatives employing RT-PCR for detection and analysis are attractive for several reasons pertaining to the COVID-19 pandemic, as they permit rapid, automated, and streamlined detection [13]. Ideally, microfluidic detection would be coupled with upstream sample preparation within one integrated device; a miniaturized total analysis system (µTAS). However, this is less easily accomplished with large volume samples, such as those encountered with large-scale pooling [14].

Pooling is a sampling method whereby multiple specimens are combined into a “batch” or “pool”, which is then analyzed using resources equivalent to those required for an individual test [15]. This serves to minimize the volumes of reagents used, lower the cost per test, increase testing capacity, and decrease the required time investment for analyses [16]. It can be especially beneficial for large-scale diagnostic screening in populations where the prevalence of the infection is low [17]. Additionally, it is a valuable tool for mitigating the spread of an illness in cases where patients are infected but asymptomatic.

Given the current steady decline in recorded cases [18], a sensitive and rapid method for this application could be essential for the efficient and accurate monitoring of infection rates. As such, a workflow for streamlined surveillance monitoring of SARS-CoV-2 with the potential for diagnosis from pooled clinical samples is detailed here. This methodology couples the ultrafast SARS-CoV-2 virus enrichment and extraction sample preparation technique optimized previously by our group with the downstream detection of pooled samples by rapid microfluidic RT-PCR [11]. For proof-of-principle, downstream detection is accomplished by conventional RT-PCR using gold-standard instrumentation and kit chemistries given Emergency Use Authorization (EUA) by the Centers for Disease Control and Prevention (CDC). Additionally, microfluidic detection from pooled eluates using a custom-built RT-PCR instrument and corresponding microchips is demonstrated [19]. The presented workflow seeks to alleviate the macro-to-micro issues associated with microfluidic testing of pooled samples in a streamlined format, thereby promoting enhanced virus surveillance and decreased transmission rates.

## 2. Materials and Methods

### 2.1. Clinical SARS-CoV-2 Sample Preparation and Analysis

Three types of clinical samples were used in this study: samples derived from nasopharyngeal swabs stored in viral transport medium (VTM), neat saliva spiked with this VTM, and saliva samples collected, transported, and stored using a DNA Genotek OMNIgene^®^ ORAL (OME-505) device (DNA Genotek Inc., Ottawa, ON, Canada). Clinical VTM samples were analyzed via real-time RT-PCR using the Abbott M2000 Real-Time SARS-CoV-2 Assay (Abbott, IL, USA) or the Xpert^®^ Xpress SARS-CoV-2 Assay (Cepheid, Sunnyvale, CA, USA) coupled with the Abbott M2000 Real-Time System (Abbott, IL, USA). Individual samples were then de-identified, assigned a sample code, and vortexed for 10 s before the transferal of a 0.6–1 mL aliquot to a pre-labeled, 2 mL screw-cap microcentrifuge tube. Aliquoted samples were inactivated via heat treatment at 65 °C for 30 min and stored in a sealed, biohazard-designated zip-top bag at –20 °C until analysis. Neat (undiluted) saliva used for VTM spiking was diluted using a diluent buffer in a 1:3 ratio before use. The diluent buffer was prepared by first dissolving 3.3073 g NaCl, 0.0807 g KCl, 0.5678 g Na2HPO4, and 0.0978 g KH2PO4 in 40 mL of molecular biology grade water (Fisher Scientific, Pittsburgh, PA, USA) to obtain a 10× phosphate-buffered saline (PBS) solution. The pH of this solution was then adjusted to 7.35 using NaOH, and 200 µL of solution was combined with 1400 µL molecular biology grade water (Fisher Scientific) and 400 µL BLUE buffer (MicroGEM International, PLC., Charlottesville, VA, USA) to create the saliva dilution buffer. Saliva samples were obtained using the DNA Genotek OMNIgene^®^ ORAL (OME-505) device for the stabilization of viral RNA in the CRL COVID-19 Self Collection Testing Kit from Clinical Reference Laboratory, Inc. (CRL, Lenexa, KS, USA). Initial COVID-19 testing conducted at CRL was performed using the CRL Rapid Response™ test with Emergency Use Authorization (EUA) granted by the FDA [20]. RNA extractions at CRL were conducted using the Zymo Quick-DNA/RNA™ Viral MagBead kit (Zymo Research Corporation, Irvine, CA, USA) on Tecan automated platforms (Tecan Life Sciences, Männedorf, Switzerland). RT-PCR was performed using the Logix Smart™ Coronavirus Disease 2019 (COVID-19) kit (Co-Diagnostics, Inc., Salt Lake City, UT, USA) using Bio-Rad CFX96™ Touch Real-Time PCR detection systems with Bio-Rad CFX Manager 3.1 software (Bio-Rad Laboratories, Hercules, CA, USA). Before receipt by the University of Virginia Health System, saliva samples were de-identified according to HIPAA standards. Samples were shipped and stored at room temperature to best approximate “real-world” conditions; the self-collection system is reported to stabilize samples at room temperature for approximately 21 days [21,22]. There was no dilution of VTM clinical samples nor self-collected saliva samples before pooling and extraction.

### 2.2. Sample Pooling Protocol

**Figure 1:**
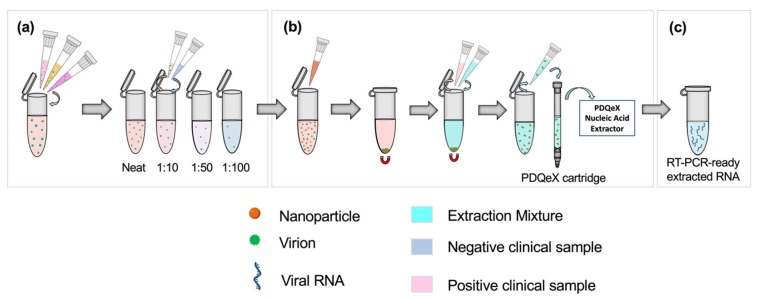
Schematic illustrating the workflow for sample preparation and RNA extraction. Based on the clinically assigned C_T_ value, samples (nasopharyngeal swabs in VTM, or saliva, as appropriate) were classified as having a high, moderate, or low viral titer (C_T_ value < 20, 20–30, or >30, respectively). (**a**) Equal volumes of three positive samples with similar clinically assigned C_T_ values were combined to represent a pooled positive sample. The appropriate ratios were achieved by diluting this pooled positive sample in clinically negative samples. (**b**) SARS-CoV-2 virions were pre-concentrated using paramagnetic nanoparticles. This step facilitated the adsorption of virions to the nanoparticles, which were then magnetically separated from the sample. The supernatant was removed, and the extraction cocktail was added for the resuspension of nanoparticles. The desorption and extraction of RNA were then facilitated via thermocycling. This process was repeated for each dilution pool. (**c**) RT-PCR-ready extracted viral RNA.

Clinical SARS-CoV-2 samples were classified as having a “high”, “moderate,” or “low” viral titer based on the clinically detected C_T_ value from real-time RT-PCR. “High”, “moderate”, and “low” samples were those assigned C_T_ values of <20, 20–30, and >30, respectively. A sample pool was created for each classification by combining 250 µL each of three positive samples with similar C_T_ values to form a 750 µL bulk sample, as shown in the first step of Figure 1a. The appropriate volumes of these positive pools were then combined with negative clinical samples to achieve 1:10, 1:50, and 1:100 dilutions of positive bulk sample to negative sample (Figure 1a). This process was repeated without deviation for all clinical sample types analyzed.

### 2.3. RNA Extractions Using the PDQeX Platform

Before extraction, samples were enriched via nanoparticle pre-concentration as described by Dignan et al. [11]. Each dilution ratio was applied to a total volume of 500 µL; for example, 50 µL of positive pooled sample was added to 450 µL of negative clinical sample for the 1:10 dilution. The dilution setup and sample preparation workflow are illustrated in Figure 1a. Briefly, 100 µL of Nanotrap^®^ Magnetic Virus Particles (CERES Nanosciences, Inc., Manassas, VA, USA) were added to each dilution pool, with thorough mixing via vortexing (Figure 1b). Per the manufacturer’s recommendation, the supernatant was removed, and the extraction cocktail was added. The cocktail comprised 88 µL of nuclease-free water, 2 µL of RNAGEM (MicroGEM US Inc, Charlottesville, VA, USA), and 10 µL of 10 X BLUE Buffer (MicroGEM, Charlottesville, VA, USA). Following thorough mixing, the combined sample and extraction cocktail mixture was transferred to a PDQeX cartridge before thermocycling in the PDQeX Nucleic acid Extractor (MicroGEM, Charlottesville, VA, USA) at 95 °C for 5 min (Figure 1b,c). This process was repeated for each dilution pool. The extracts were then immediately analyzed via RT-PCR.

### 2.4. RT-PCR Conditions

The C_T_ data obtained from RT-PCR experiments were used to evaluate the relative success of upstream preparation and detection of SARS-CoV-2 viral RNA in pooled and diluted samples. The RT-PCR assay used was developed by the Centers for Disease Control and Prevention (CDC) under an emergency use authorization (EUA) in February 2020 [23]. Each reaction was a total volume of 20 µL and was comprised of 5 µL of viral RNA extract, 5 µL of TaqPath™ 1-Step RT-q-PCR Master Mix (Thermo Fisher Scientific, Waltham, MA, USA), 1 µL of SARS-CoV-2 (2019-nCoV) CDC RUO N1 primer-probe mix (Integrated DNA Technologies, Coralville, IA, USA), and 9 µL of PCR-grade water. The 2019-nCoV_N_Positive Control plasmid (Integrated DNA Technologies, Coralville, IA, USA) was serially diluted to obtain concentrations of 1000, 100, 50, and 10 copies/µL and used as positive controls for real-time RT-PCR. Samples were run in either triplicate or quintuplicate using the suggested protocol for the TaqPath™ 1-Step RT-q-PCR Master Mix, including UNG incubation at 25 °C for 120 s, reverse transcription at 50 °C for 900 s, polymerase activation at 95 °C for 120 s, and 40 amplification cycles (95 °C for 3 s and 60 °C for 30 s). Extracts and controls were analyzed using a QuantStudio™ 5 Real-Time PCR System for Human Identification (Applied Biosystems, Waltham, MA, USA).

### 2.5. Double-Blind Study—Clinical Saliva Samples

A double-blind study was conducted to assess the accuracy of qualitative differentiation between clinical positive and negative samples using the proposed pooling protocol while ensuring the absence of selection bias. Deidentified clinical negative and positive saliva samples (obtained using the OMNIgene^®^•ORAL self-collection system) were randomly selected, analyzed via RT-PCR, and labeled with a numerical sample code at Clinical Reference Laboratory, Inc. Samples were then shipped to the researchers at UVA for analysis. Each sample was then randomly assigned an in-house code (X1–X10) which correlated to the numerical sample code provided by the third party. These samples were diluted to the appropriate ratios using negative saliva samples, as detailed in Figure 1a, before RNA extraction and RT-PCR analysis (Figure 1b,c). Presumptive results were then conveyed to the third party for comparison to known data.

### 2.6. Adaptation of Assay to an Ultra-Rapid Real-Time Microfluidic PCR Amplification Instrument

Preliminary experiments were conducted using clinical saliva samples to determine the feasibility of adapting the assay for use with a novel in-house PCR amplification instrument. Data from the in-house device were compared to conventional gold-standard techniques. Extraction was performed using the RNeasy Mini Kit (Qiagen, Valencia, CA, USA), and RT-PCR was performed utilizing the QuantStudio™ 5 Real-Time PCR System for Human Identification and the manufacturer’s thermocycling protocol as previously described. Experiments were conducted in parallel using the PDQeX Nucleic Acid Extractor with Nanotrap^®^ Magnetic Virus Particles enrichment for extraction, while PCR amplification was realized using the Ultra-Rapid Real-Time Microfluidic PCR Amplification instrument. TaqPath™ 1-Step RT-q-PCR Master Mix and SARS-CoV-2 (2019-nCoV) CDC RUO N1 primer-probe mix chemistry was used for both conventional and microfluidic RT-PCR.

Briefly, 300 µL was taken from each of four positive clinical saliva samples and combined to create a neat pool with an average reported C_T_ value of 21.7 ± 1.06 and a total volume of 1.2 mL. A 1:50 dilution pool was prepared from the amalgamated neat stock using negative saliva samples as diluent with a final volume of 600 µL. The neat and 1:50 pools were split equally, with half designated for conventional analyses and the remainder for use in microfluidic experiments. Then, 250 µL each was taken from each pool and analyzed using conventional methods. Identical aliquots were analyzed using the microfluidic method described above. Negative saliva samples were included as controls, and the conventional RT-PCR was conducted according to the manufacturer’s protocol as previously described. For microfluidic experiments, however, the thermocycling program comprised reverse transcription at 50 °C for 300 s, polymerase activation at 95 °C for 120 s, and 40 amplification cycles (95 °C for 1 s and 60 °C for 15 s). Data obtained were analyzed and compared.

### 2.7. Statistical Analyses

All statistical analyses were conducted using GraphPad Prism version 9.3.1. (350) for macOS, GraphPad Software, San Diego, CA, USA [24].

## 3. Results

### 3.1. Pooling of Nasopharyngeal (VTM) Clinical Sample

**Figure 2:**
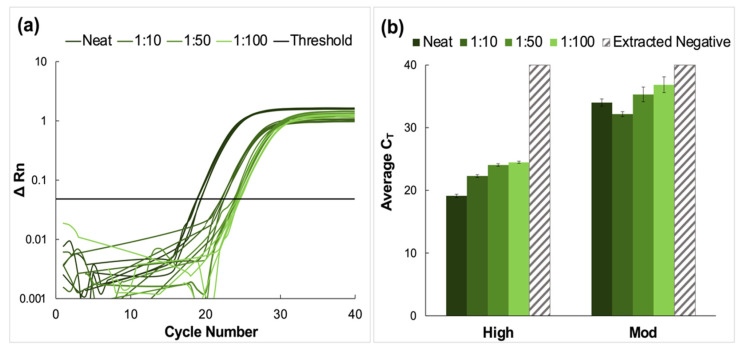
Amplification data obtained from pooling experiments using clinical nasopharyngeal swab samples. (**a**) A representative normalized amplification plot obtained from RT-PCR analysis of the extracted RNA (high viral titer). (**b**) Summary of CT data. Dilution ratios increase from left to right. Amplification was successful for all dilutions of high and moderate viral titer clinical samples (*n* = 5). Tukey’s multiple comparisons tests showed that in the case of high viral titer clinical samples, there was no significant statistical difference between CT data obtained for the neat and 1:10 pools (*p* > 0.9999). However, significant differences were obtained when the neat was compared to 1:50 and 1:100 dilutions (*p* < 0.0001, in both cases), when the 1:10 pool was compared to 1:50 and 1:100 dilutions (*p* < 0.0001, in both cases), and when the 1:50 and 1:100 pools were compared (*p* = 0.0196). For moderate titer samples, there was no significant difference between the average CT values obtained for neat samples compared to those obtained for 1:10 and 1:50 dilutions (*p* = 0.2040 and 0.0954, respectively). However, statistical differences were observed when the neat vs. 1:100, 1:10 vs. 1:50, 1:10 vs. 1:100, and 1:50 vs. 1:100 pools were compared (*p* < 0.0001, 0.0016, <0.0001, and 0.0136, respectively).

Clinical nasopharyngeal (NP) samples classified as having ‘high’ and ‘moderate’ relative viral titers were pooled and prepared using the nanoparticle enrichment and enzymatic extraction protocol. A representative amplification plot from the RT-PCR analysis of extracted RNA from the high titer pool and the corresponding dilutions of this sample type is shown in Figure 2a. The average C_T_ values for high and moderate pools are summarized in Figure 2b. Amplification was observed for all dilutions in these sample pools. The average C_T_ values for high titer samples increased incrementally from 19.1 ± 0.27 for neat samples to 24.5 ± 0.17 for the most dilute pool (1:100). Tukey’s multiple comparisons tests showed that for these high titer samples, there was no significant statistical difference between data obtained for the neat samples and those for the 1:10 dilution (*p* > 0.9999). Still, significant differences were observed for neat samples compared to 1:50 and 1:100 dilutions (*p* < 0.0001, in both cases). Additionally, significant statistical differences were observed when the 1:10 dilutions were compared to 1:50 and 1:100 pools (*p* < 0.0001, in both cases) and when the 1:50 pool was compared to the 1:100 pool (*p* = 0.0196).

Average C_T_ values for moderate viral titer dilutions increased from 34.0 ± 0.58 for the neat samples to 39.4 ± 1.26 for the 1:100 dilution. For these samples, there was no significant difference between the average C_T_ values obtained for neat samples compared to those obtained for 1:10 and 1:50 dilutions (*p* = 0.2040 and 0.0954, respectively). However, statistical differences were observed when the neat vs. 1:100, 1:10 vs. 1:50, 1:10 vs. 1:100, and 1:50 vs. 1:100 pools were compared (*p* < 0.0001, 0.0016, <0.0001, and 0.0136, respectively) [24,25]. No amplification was observed in the extracted negative samples, and these were assigned the maximum cycle value (40).

### 3.2. Spiking of Negative Saliva and Subsequent Pooling

**Figure 3:**
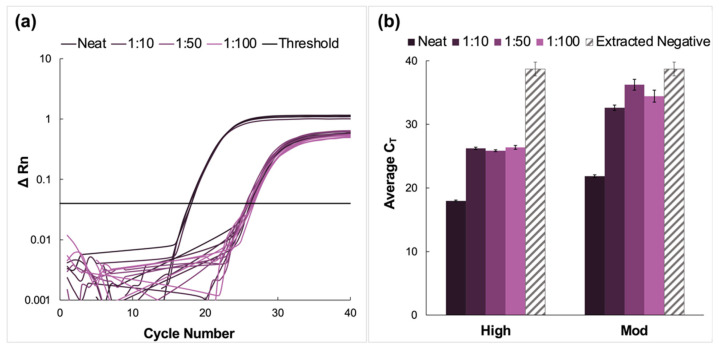
Amplification data obtained from pooling experiments using fresh saliva spiked with clinical nasopharyngeal swab samples. (**a**) A representative normalized amplification plot obtained from RT-PCR analysis of the extracted RNA (high viral titer). (**b**) A summary of the CT data. Dilution ratios increase from left to right. In this case, there was amplification in extracted negative samples (*n* = 5). For high viral titer dilution pools, Tukey’s multiple comparison test determined that although the data obtained for the 1:10 and 1:100 dilutions were statistically different (*p* < 0.0001), there was no significant difference between the average CT values obtained for the 1:50 compared to the 1:100 dilutions (*p* = 0.7520). There were also no statistically significant differences when neat samples were compared to the dilution pools (*p* < 0.0001), nor for 1:10 vs. 1:50 and 1:100 dilutions (*p* = 0.0008 and <0.0001, respectively). The average CT values for all moderate dilution pools were found to be statistically different when compared using Tukey’s test (*p* < 0.0001 for neat vs. all dilutions and 1:10 vs. 1:50 dilution pools. *p* = 0.0152 for 1:10 vs. 1:100 pools, and 0.0171 for 1:50 vs. 1:100 pools).

Here, clinical NP samples of high and moderate concentrations were spiked into fresh saliva, pooled, and analyzed via the previously described protocol. A representative amplification plot from the RT-PCR analysis of extracted RNA from the high titer pool and the corresponding dilutions of this sample type is shown in Figure 3a. The average C_T_ values for high and moderate pools are summarized in Figure 3b. There was successful amplification for all dilutions and viral titer designations following RT-PCR. Additionally, amplification was observed in the extracted negative samples, with an average C_T_ value of 38.7 ± 1.08.

For high viral titer samples, there was an increase in C_T_ value when the dilution factor increased from 1:10 to 1:50 and a decrease for the 1:100 pool. Still, Tukey’s multiple comparison test determined that although the data obtained for the 1:10 and 1:100 dilutions were statistically different (*p* < 0.0001), there was no significant difference between the average C_T_ values obtained for the 1:50 compared to the 1:100 dilutions (*p* = 0.7520) [24,25]. There were also no statistically significant differences when neat samples were compared to the dilution pools (*p* < 0.0001) nor for 1:10 vs. 1:50 and 1:100 dilutions (*p* = 0.0008 and < 0.0001, respectively).

In the case of the moderate C_T_ samples, there was a significant increase in average C_T_ values for the diluted samples compared to the neat, from 21.9 ± 0.42 for the neat sample up to 34.4 ± 0.30 for the 1:100 dilution. This was followed by a rise in C_T_ values for the 1:50 dilution before a decrease in average C_T_ values for the 1:100 dilutions. Unlike the high-titer samples, the average C_T_ values for all moderate dilution pools were found to be statistically different when compared using Tukey’s test (*p* < 0.0001 for neat vs. all and 1:10 vs. 1:50 dilution pools. *p* = 0.0152 for 1:10 vs. 1:100, and 0.0171 for 1:50 vs. 1:100 pools) [24,25].

### 3.3. Pooling of Saliva Samples

**Figure 4:**
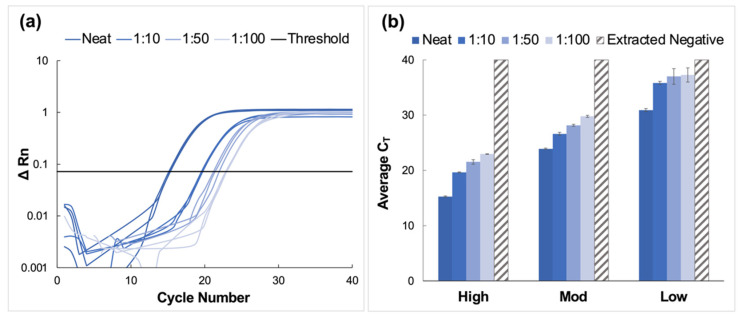
Amplification data obtained from pooling experiments using saliva samples collected using the CRL OMNIgene^®^•ORAL sampling kit. (**a**) A representative normalized amplification plot obtained from RT-PCR analysis of extracted RNA (high viral titer). (**b**) Summary of CT data. Dilution ratios increase from left to right (*n* = 3). It was determined using Tukey’s test that the average CT values for the high viral titer dilutions were statistically distinct from each other (*p* < 0.0001). This was also true for the moderate viral titer pools (*p* < 0.0001). However, for low viral titer dilutions, it was determined that there was no statistical difference between means when the 1:50 dilutions were compared to those diluted to 1:100 (*p* > 0.9999). For all other dilutions, statistical differences were significant (*p* < 0.0001 for neat vs. all dilutions, and *p* = 0.0285 and 0.0243 for 1:10 vs. 1:50 and 1:100 dilutions, respectively.

High, moderate, and low concentration clinical saliva samples obtained using the CRL COVID-19 Self Collection Testing Kit were pooled and prepared as described above. Amplification was observed for all samples and corresponding dilutions (Figure 4).

C_T_ values increased by approximately four units for each viral titer (high, moderate, and low) when comparing neat samples to those diluted to the corresponding 1:10 dilutions; however, the deltas of the average C_T_ values decreased for dilutions beyond 1:10. Namely, C_T_ values increased by approximately 1.6 cycles between dilutions for high and moderate samples and between 0.27 and 1.2 cycles for low concentration samples. Overall, the average C_T_ value for low-titer samples incrementally increased from 30.9 ± 0.28 for the neat sample to 37.3 ± 1.29 for the 1:100 dilution.

Average C_T_ values ranged from 23.9 ± 0.13 for the neat sample to 29.8 ± 0.19 for the 1:100 dilution for moderate viral titer samples. As seen with the high titer samples, there was a consistent incremental increase in average C_T_ value as the negative to positive clinical sample ratio increased. It was determined using Tukey’s test that the average C_T_ values for the high viral titer dilutions were statistically distinct from each other (*p* < 0.0001) [24,25]. This was also true for the moderate viral titer pools (*p* < 0.0001). However, for low viral titer dilutions, it was determined that there was no statistical difference between means when the 1:50 dilutions were compared to those diluted to 1:100 (*p* > 0.9999). For all other dilutions, statistical differences were significant (*p* < 0.0001 for neat vs. all dilutions, and *p* = 0.0285 and 0.0243 for 1:10 vs. 1:50 and 1:100 dilutions, respectively).

### 3.4. Double-Blind Study—Clinical Saliva Study

Clinical saliva samples obtained using CRL COVID-19 Self Collection Testing Kits were first analyzed at CRL, blinded, and then transferred to our lab to facilitate the double-blind study. Pooling, enrichment, and extractions were conducted in the same manner as described for samples of known viral concentrations. The presumptive qualitative diagnoses (positive or negative) for dilution pools created using each of 10 blinded samples were 100% concordant with clinical results; five positive and five negative samples were accurately identified using the optimized pooling protocol. No amplification was observed in the samples which were later confirmed as negative.

**Figure 5:**
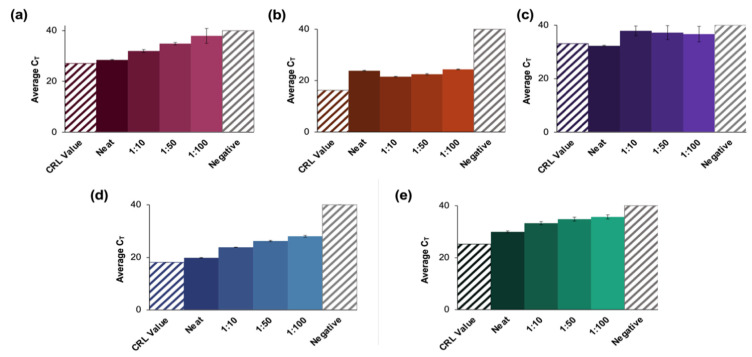
Results of a blind study conducted using CRL clinical saliva samples. (**a**–**e**) summarize the data obtained from evaluations of blinded CRL samples. Pools for each of 10 blinded samples were created and tested according to the developed protocol and were found to be in 100% agreement with the reported qualitative results (five positive and five negative samples). The significant increase in CT value obtained for (**b**,**e**) could be attributed to collection at an earlier date than other positive samples. These may thus have had a lower viral load when analyzed than at initial CRL testing. A negative clinical saliva sample was extracted according to the protocol described for positive clinical samples. (**a**–**e**) represent X2, X5, X7, X8, and X9, respectively, and *n* = 3 in each case. For X2 dilutions, there were no significant differences between the neat samples vs. the 1:10 dilutions (*p* = 0.0931) or the CRL reported value (*p* > 0.9999). There were also no statistical differences between the data for the 1:50 dilutions vs. 1:100 dilutions (*p* = 0.0329). For X5 dilutions, the reported value was statistically different from the average CT value for the neat samples (*p* < 0.0001), but there was no significant difference between values obtained for the neat samples vs. the 1:100 dilutions. For X7 dilutions, the only statistically distinct groups were the neat samples vs. 1:10 dilutions (*p* = 0.0441). For X8 dilutions, all pools were statistically different (*p* < 0.0001), and for X9, the 1:10 vs. 1:50, and the 1:50 vs. 1:100 dilutions were statistically similar (*p* = 0.1058 and 0.5762, respectively).

The amplification results for positive samples pooled up to 1:100 are shown in Figure 5, along with the C_T_ value obtained from CRL following initial analyses at their location (depicted as a colored, shaded bar).

For sample X2 (Figure 5a), the average C_T_ values obtained for the neat samples were ≈1.5 cycles higher than the reported CRL value (27.1) and ranged from 28.5 ± 0.33 for the neat sample up to 33.3 for the 1:100 dilution (*n* = 3). Statistical analyses of X2 dilutions determined that there were no significant differences between the neat samples vs. the 1:10 dilutions (*p* = 0.0931) or the CRL reported value (*p* > 0.9999). Additionally, there were no statistical differences between the data obtained for the 1:50 dilutions compared to the 1:100 dilutions (*p* = 0.0329) [24,25].

In the case of sample X5 (Figure 5b), C_T_ values increased from a reported value of 16.2 to 23.8 ± 0.19 for the neat sample up to 24.3 ± 0.16 for the 1:100 dilution. Although there was a general overall increase in average C_T_ values as the dilution factor increased, the neat samples had a higher average C_T_ value than the 1:10 dilution. Additionally, the reported value was statistically different from the average C_T_ value for the neat samples (*p* < 0.0001) in this case [24,25]. There was no significant difference between the value obtained for the neat samples vs. the 1:100 dilutions.

For sample X7 (Figure 5c), there was no apparent trend regarding changes in average C_T_ values as dilution ratios changed. The only statistically distinct groups were the neat samples compared to the 1:10 dilution (*p* = 0.0441) [24,25]. There were also more significant standard deviations observed in this group than seen in any other analyses of blind samples. The average C_T_ values increased as the dilution ratio increased for samples X8 and X9 (results summarized in Figure 5d,e, respectively). For X8, values ranged from 19.8 ± 0.13 to 28.0 ± 0.36 as dilution ratios increased (CRL value = 18.1), while the average C_T_ values ranged from 29.9 ± 0.36 to 35.7 ± 0.79 for sample X9 (CRL value = 25.2). Additionally, for X8 dilutions, all pools were found to be statistically distinct (*p* < 0.0001). Statistical analyses showed that for sample X9, the 1:10 dilutions vs. 1:50, as well as the 1:50 vs. 1:100 were not significantly different (*p* = 0.1058 and 0.5762, respectively).

### 3.5. Integration of Assay into a Microfluidic Format

**Figure 6:**
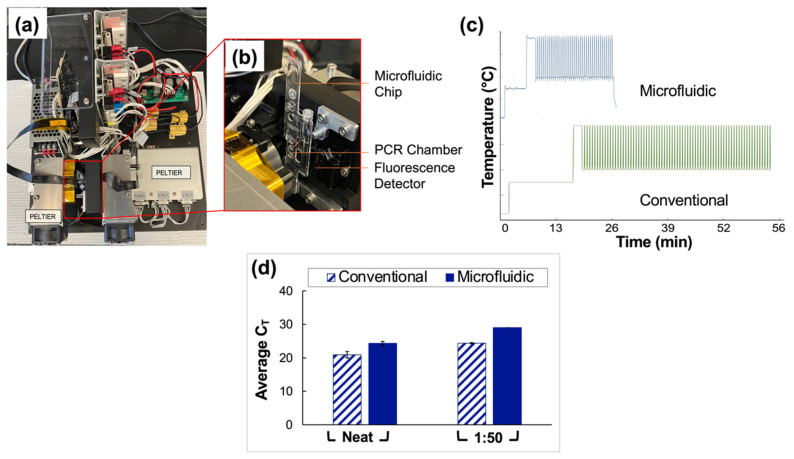
Comparison of conventional and microfluidic methods—Qiagen extraction and QuantStudio 5 RT-PCR vs. PDQeX extraction and amplification with ultra-rapid instrument, respectively. (**a**) Top view of the microfluidic instrument used for rapid PCR analyses. (**b**) Magnified and annotated view of the microfluidic chip as typically placed in the instrument for analyses. Before use of the device, this chip is sandwiched securely between two Peltiers to facilitate thermocycling, and the instrument is covered with a light-proof box. (**c**) Temperature profile expected for the conventional instrument (63 min runtime) compared to that obtained via thermocouple monitoring for the microfluidic instrument (25 min runtime). (**d**) Summary of data obtained from a direct comparison of conventional and microfluidic methods (Qiagen extraction and QuantStudio 5 RT-PCR vs. PDQeX extraction and amplification using the ultra-rapid instrument, respectively. *n* = 3 in each case). The average C_T_ values acquired using the conventional vs. microfluidic methods were statistically different (as determined by *t*-tests, *p* = 0.0060 for comparison of neat samples and <0.0001 for 1:50 dilutions).

Clinical saliva samples were analyzed using conventional vs. microfluidic methods, and the results were compared. The portable, in-house ‘Ultra-Rapid Real-Time Microfluidic PCR Amplification’ instrument used for the microfluidic analyses is shown in Figure 6a,b. Following extraction and RT-PCR analyses, amplification was observed in all reported positive samples tested for neat and 1:50 pools using both conventional and microfluidic methods. The average C_T_ values obtained for the traditional assays were 20.9 ± 0.95 and 24.4 ± 0.16 for neat and 1:50 sample types, respectively. Microfluidic analyses yielded C_T_ values of 24.3 ± 0.58 and 29 (no deviation) for neat and 1:50 sample types.

Additionally, the temperature profile obtained using a thermocouple to monitor the progression of thermocycling in the microfluidic chip chamber is compared to that projected for the conventional instrument in Figure 6c. The microfluidic device consistently and accurately achieved target temperatures of 95 °C and 60 °C while significantly reducing the overall assay runtime from 63 to 27 min. An increase of 3.5 units for the average C_T_ values of the neat pools was obtained microfluidically compared to conventional methods. Additionally, there was an increase of 4.7 units when data for the 1:50 pools obtained using microfluidic methods were compared to conventional methods. A graphical comparison of C_T_ values obtained using conventional vs. microfluidic methods is given in Figure 6d.

## 4. Discussion

A previously reported protocol for ultrafast viral enrichment and enzymatic extraction was used for upstream sample preparation from pooled SARS-CoV-2 samples in multiple sample matrices [11]. Here, viral transport media derived from nasopharyngeal samples, fresh saliva spiked with VTM from nasopharyngeal samples, and saliva samples obtained using a commercially available collection system spanning commonly observed C_T_ values were analyzed using the adapted protocol, which leveraged magnetically actuated nanoparticles to capture and isolate virions from the clinical sample matrices.

To demonstrate the sensitivity and performance of the adapted protocol, average C_T_ values acquired from RT-PCR of prepared viral RNA were compared. Relative viral concentrations and potential effects of large-scale dilutions were evaluated based on the theory that any adverse effects arising from sample preparation or dilution steps would result in slower or decreased amplification and comparatively increased C_T_ values. It was expected that an increase in dilution factor would result in a corresponding rise in average C_T_ value for dilutions within sample pools [26]. This general trend of a commensurate increase in C_T_ value and dilution factors reflects a synchronous reduction in the number of virions present in the overall volume. Here, we demonstrate the successful enrichment, extraction, and detection of virions with minimal loss of amplification or sample concentration with the proposed workflow.

### 4.1. Pooling of Nasopharyngeal (VTM) Samples

Pooling studies were initially completed with VTM nasopharyngeal samples, as previous experiments had demonstrated the successful application of the method with clinical samples of this type [11]. Here, dilutions of 1:10, 1:50, and 1:100 were evaluated; although it was unlikely that pools of 1:50 or 1:100 will be utilized in practice, it was deemed critical to assess the limits of the method as a measure of its large-scale applicability.

Neat VTM samples classified as having a high viral load (reported C_T_ value < 20) were successfully detected from samples diluted up to 1:100. A general increase in C_T_ value was observed for samples classified as moderate except for the 1:10 dilution, where replicate C_T_ values were lower than that of the neat sample. Although this was unexpected, statistical analyses (Tukey’s multiple comparisons tests) found no significant difference between these means, as described in the Results section. It was determined that the unexpected occurrence was not prohibitively detrimental to the comprehensive analysis of the data set. Samples reported as having low viral loads were excluded from these analyses, as previous experiments showed that for samples of this type, as C_T_ values approached the maximum number of cycles (40, in this case), it was more likely that the RNA was not homogeneously distributed; consequently, the distribution of data points was abnormal [11].

### 4.2. Pooling of Neat Saliva Spiked with VTM Clinical Samples

When clinical NP samples were spiked with fresh saliva, pooled, and prepared via the previously described protocol, successful amplification was observed for high and moderate viral titer samples. Yet, the theorized trend whereby C_T_ value was expected to increase incrementally as the dilution factor increased was not evident. Previous work demonstrated no significant difference in data obtained using diluted clinical VTM samples compared to saliva diluted with an in-house diluent buffer [11]. However, in that case, preliminary experiments only investigated dilutions up to 10×. The data reported here indicate somewhat stochastic deviations from the expected trend at higher dilutions. Presumably, as the ratio of fresh saliva to sample increased, the inhomogeneity of sample matrices, that is, VTM compared to saliva, induced disparate virion responses to sample enrichment. Although alleviated by the addition of buffer solution, the viscosity of saliva also contributed to the inhomogeneity of samples, as mixing was likely inefficient.

Additionally, this was the only sample type where the amplification of extracted negatives was observed. Although the average C_T_ values approximated 40 cycles, this was a nonideal occurrence and could likely be attributed to experimental contamination or, potentially, the detection of a noninfectious, weak positive. It should be noted that the saliva used was obtained from volunteers who had recently (within 3–5 days before donation) tested negative for the presence of the virus. Consequently, while the spiking of VTM into fresh saliva served to approximate workflow performance with pooled clinical saliva samples, the challenges associated with this sample type further validated the need to test uncontrived saliva samples.

### 4.3. Pooling of Clinical Saliva Samples

The CRL COVID-19 Self Collection Testing kit, which includes the DNA Genotek OMNIgene^®^ ORAL (OME-505) device, was granted Emergency Use Authorization (EUA) by the FDA in 2020 based on extensive trials and favorable results from performance evaluations [20]. Consequently, this was deemed an ideal source of clinical saliva samples, which is reflective of existing pandemic control measures.

Average C_T_ values increased stepwise for all clinical saliva samples, especially for high and moderate viral titer samples. This observation was attributed to slight decreases in sensitivity as the negative to positive sample ratio increased. The increase was less consistent for low-viral titer dilutions, with the samples of higher dilution ratios statistically approximating the cycle numbers for extracted negative samples. This observation was not extraordinary, given the low virion concentration and the potential for associated stochastic sampling. Nonetheless, qualitative detection in clinical samples with very low viral loads was successfully demonstrated. Additionally, previous studies have indicated that the likelihood of infectivity for patients whose samples are in this range is extremely low, thereby minimizing the potential adverse effects that could result from similarly ambiguous results [27].

The acquisition of more conclusive results with this sample type when compared to data obtained for contrived saliva samples may also indicate that the buffering system used plays a critical role in determining the feasibility of using saliva samples for diagnoses; the commercially available test kit and the associated reagents appeared to better stabilize and homogenize these samples than the in-house buffering solution, leading to more facile downstream analysis.

### 4.4. Double-Blind Study—Clinical Saliva Samples

The presumptive qualitative diagnoses obtained from pooled saliva samples diluted as high as 1:100 from blinded samples were 100% in agreement with the reported clinical results. Furthermore, examination of the results from pooled samples shows that generally, as the dilution factor increased, so did the C_T_ value. Beyond concordance, the values obtained from these analyses were compared with those obtained from CRL; it was hypothesized that differences in C_T_ values would occur as sample preparation, extraction, RT-PCR, and experimental conditions were not identical. There was no statistical difference between the values we obtained for neat samples and those reported by CRL for samples X2, X7, or X8 (Figure 5a,c,d). However, dissimilarities were pronounced for samples X5 and X9 (Figure 5b,e), for which C_T_ values increased by 7.4 and 4.4 units, respectively. Interestingly, when results were compared to clinical data to determine the degree of concordance, CRL reported that these samples were several days older than the other eight blinded samples and, as such, may have been adversely affected by sample degradation. This explains the disparity in average C_T_ value; the initial viral load detected by CRL likely decreased during the elapsed period, while additional samples were accumulated, blinded, subsequently shipped, and finally analyzed by the described method.

Despite fluctuations in average C_T_ values for X5 and X7 (Figure 5b,c), successful qualitative diagnoses were achieved using the described pooling protocol. For sample X7 specifically, these differences could be explained by the low initial viral load indicated by C_T_ values of 33.1 (CRL) and 32.3 ± 0.20 (experimental values). As seen with low-viral titer saliva samples in non-blinded experiments, these results can likely be attributed to stochastic sampling.

### 4.5. Integration of Assay into a Microfluidic Platform

Given the successful application of the described pooling method to assays conducted using a gold-standard RT-PCR instrument, namely the QuantStudio™ 5 Real-Time PCR System for Human Identification, the next phase of experiments will entail optimization of the method for a novel portable, in-house ‘Ultra-Rapid Real-Time Microfluidic PCR Amplification’ instrument, as shown in Figure 6a,b [19]. Preliminary results shown here (Figure 6c) indicate that the device is capable of decreasing the overall runtime for the assay from approximately 63 to 27 min (a total time reduction of about 57%) when the assay, currently under optimization, is compared to the manufacturer’s (TaqPath™ 1-Step RT-q-PCR Master Mix) recommended protocol. Additionally, experiments indicate that this runtime can be reduced further. This reduction was achieved by varying thermocycling conditions, for example, ramp rates and dwell times, to determine the feasibility of acquiring satisfactory data while using conditions other than those outlined by the manufacturer. Figure 6d illustrates the results obtained when conventional methods—gold standards for extraction and RT-PCR (Qiagen and QuantStudio 5)—are compared to those obtained using the microfluidic platform. When neat and 1:50 dilutions of clinical saliva sample pools were analyzed in parallel, the average C_T_ values acquired using the conventional vs. microfluidic methods were statistically different (as determined by *t*-tests, *p* = 0.0060 for comparison of neat samples and <0.0001 for 1:50 dilutions). However, the potential for a substantial reduction in total runtime, the eventual goal of creating a fully integrated portable instrument, and the demonstrated successful qualitative analyses of up to 1:50 dilutions of clinical saliva samples serve to temper the nonideal increase in cycle numbers. This instrument also introduces the potential for rapid, point-of-need (PON), qualitative diagnosis of pooled samples in large-scale settings pending the projected integration of extraction and amplification stages into a single miniaturized total analysis system.

## 5. Conclusions

The application of an enrichment and extraction method to large-scale diagnostic screening for SARS-CoV-2 is described herein. Experimental results indicate that the technique is compatible with pooled samples up to dilutions of 1:100 and with a range of sample types, including neat VTM eluate from NP swabs and saliva samples stored in DNA Genotek’s proprietary buffer solution for the OMNIgene^®^ ORAL (OME-505) device. Additionally, the successful detection of SARS-CoV-2 was achieved for these sample types with minimal loss in sensitivity and comprehensive qualitative accuracy. Still, in the case of contrived saliva samples prepared by spiking NP-derived VTM into fresh saliva, the results obtained were inconclusive and inconsistent, suggesting that the in-house buffering solution used for homogenization and stabilization of the saliva matrix requires further optimization to perform comparably to commercial test kits. This is further substantiated by the fact that the average C_T_ values obtained from our analyses of the saliva samples collected using these kits approximated those from the third party despite undergoing shipment and multi-day storage.

A double-blind study was also conducted to objectively investigate the performance of this method when applied to “real world” use. The results of this study which utilized the proposed method were 100% concordant with the reported clinical values, with the accurate qualitative designation of five positive and five negative samples achieved, suggesting the potential for obtaining accurate clinical diagnoses in clinical settings.

Finally, preliminary experiments demonstrated the potential for the optimization of the pooling assay for point-of-need (PON) deployment using a novel, ultra-rapid, microfluidic PCR amplification device. Future work entails further optimizing this assay to determine the shortest runtime possible without a significant decrease in the accuracy and sensitivity of diagnoses and eventual integration of the optimized amplification and all sample preparation steps into a portable miniaturized total analysis device.

## Data Availability

The data presented in this study are available on request from the corresponding author. The data are not publicly available due to privacy concerns.
